# 18 month audit of anesthetist led sedation vs podiatric ankle and poplitieal blocks: review of protocols, procedures and patient perceptions

**DOI:** 10.1186/1757-1146-3-S1-O4

**Published:** 2010-12-20

**Authors:** Ian Beech

**Affiliations:** 1 NHS Wandsworth, London, UK

## 

A review of 100 patients between January 2009 - June 2010 undergoing foot surgery requiring an ankle or popliteal block were assessed on their personal perceptions of the injection procedure in terms of expectations of pain and intra-surgical experience. Comparative results are made with those who had anaesthetist led sedation and an ankle block only. Preliminary results indicate those patients selected/elected for the local anaesthetic had a favourable experience, the majority would be happy to repeat their procedure under similar conditions. Sedation patients had a very similar patient experience profile. This small study supports the view that good practice for Podiatric Surgical patients is to offer an anaesthetist led sedation or general anaesthesia option but it is not essential or desirable for all patients.

